# Emerging Infections Program—State Health Department Perspective

**DOI:** 10.3201/eid2109.150428

**Published:** 2015-09

**Authors:** James L. Hadler, Richard N. Danila, Paul R. Cieslak, James I. Meek, William Schaffner, Kirk E. Smith, Matthew L. Cartter, Lee H. Harrison, Duc J. Vugia, Ruth Lynfield

**Affiliations:** Yale School of Public Health, New Haven, Connecticut, USA (J.L. Hadler, J.I. Meek);; Minnesota Department of Health, St. Paul, Minnesota, USA (R.N. Danila, K.E. Smith, R. Lynfield);; Oregon Health Authority, Portland, Oregon, USA (P.R. Cieslak);; Vanderbilt University School of Medicine, Nashville, Tennessee, USA (W. Schaffner);; Connecticut Department of Public Health, Hartford, Connecticut, USA (M.L. Cartter);; Johns Hopkins Bloomberg School of Public Health, Baltimore, Maryland, USA (L.H. Harrison);; California Department of Public Health, Richmond, California, USA (D.J. Vugia)

**Keywords:** surveillance, infectious diseases emerging, laboratories, education, training, capacity building, academic medical center emerging infections programs, antimicrobial resistance, bacteria, enteric infections, influenza, parasites, respiratory infections, rickettsia, staphylococci, streptococci, vector-borne infections, viruses, zoonoses, EIP, Emerging Infections Program

## Abstract

The Emerging Infections Program (EIP) is a collaboration between the Centers for Disease Control and Prevention and 10 state health departments working with academic partners to conduct active population-based surveillance and special studies for several emerging infectious disease issues determined to need special attention. The Centers for Disease Control and Prevention funds the 10 EIP sites through cooperative agreements. Our objective was to highlight 1) what being an EIP site has meant for participating health departments and associated academic centers, including accomplishments and challenges, and 2) the synergy between the state and federal levels that has resulted from the collaborative relationship. Sharing these experiences should provide constructive insight to other public health programs and other countries contemplating a collaborative federal–local approach to collective public health challenges.

## Abstract 2

This network has enriched research and workforce training and produced a synergy between state and federal levels.

In 1994, the Centers for Disease Control and Prevention (CDC) created the domestic Emerging Infections Program (EIP) as part of the response to the 1992 Institute of Medicine report recommending “the development and implementation of strategies that would strengthen state and federal efforts in U.S. surveillance” (*1*,*2*). The EIP was established as a collaborative population-based surveillance program involving CDC, selected state health departments, and their chosen academic institution partners. The major objective of the EIP was to conduct active population-based surveillance for a range of domestic emerging infectious diseases for which either no surveillance was occurring or state-level surveillance was occurring but “gold standard,” consistently high-quality surveillance was needed. The selected state health departments needed to engage clinical laboratories and infection control professionals throughout their jurisdictions. The relationship between CDC and the state health departments chosen to foster the EIP objectives has been a collaborative one, not purely a contractual relationship. Using a cooperative agreement funding mechanism, the federal, state, and academic collaborators have had shared responsibilities for setting priorities, planning and executing activities, and synthesizing and communicating results (*3*).

The infrastructure and expanded capacity that has resulted in terms of resources and collaborative relationships with CDC, between sites, and within each participating state have greatly enriched public health practice at each site and provided multiple state-based “laboratories” to pilot a variety of surveillance initiatives with possible national public health implications. The results have been remarkable: data to drive local and national public health initiatives have been gathered; state laboratory capacity to support surveillance has been updated and expanded, providing a model for expansion in other states; health threats from emerging infectious diseases have been identified and brought to national attention, and their epidemiology has been described; new methods to conduct surveillance have been piloted and adopted; staff in academic centers have become involved in public sector public health practice and research and expanded on them; and training and practice opportunities for public health students—the future epidemiology workforce—have multiplied.

In this article, our objectives are to describe 1) highlights of what being an EIP site has meant for participating health departments and associated academic centers, including accomplishments and challenges, and 2) the synergy between the state and federal levels that has resulted from the collaborative relationship. We hope that sharing these experiences will provide constructive insights to other public health program areas and other countries that are contemplating a collaborative national–local approach to collective public health challenges.

## Health Department Infrastructure and Surveillance Enhancements

Several critical state-level, surveillance-related infrastructural enhancements have resulted from being an EIP site. First, federal EIP funding has been substantial. In 2014, EIP sites received an average of $3.6 million for personnel (including indirect costs), laboratory support, and supplies for all EIP projects in which they participated. This funding paid for a range of staff members, from 22 full-time equivalent (FTE) persons (spread over 27 positions) in the site with the smallest amount of personnel support to 58 FTEs (spread over 80 positions) in the site with the highest level of personnel support. The FTEs included staff in collaborating academic centers but excluded students in training positions.

Having additional epidemiology staff made it possible to conduct gold-standard surveillance for all diseases of EIP interest, with routine auditing of laboratories becoming an accepted feature of laboratory surveillance, thereby ensuring as close to 100% reporting from laboratories as possible. The experience and contacts from these efforts have made it possible for those running programs for non-EIP diseases (e.g., HIV, tuberculosis, sexually transmitted infections) to incorporate audits into their surveillance activities.

The additional resources, also made possible expansion of laboratory capacity to support surveillance. Additional staff enabled processing and storage of specimens of organisms from persons with invasive pneumococcal disease, group A *Streptococcus* (GAS) disease, and bacterial foodborne illness to enable typing and antimicrobial susceptibility testing, critical to the expanded surveillance role EIP sites have served for these infections. Updated laboratory capacity to perform pulsed-field gel electrophoresis enabled the EIPs to be in the forefront of identifying and investigating foodborne pathogen clusters and methicillin-resistant *Staphylococcus aureus* (MRSA) strains (*4*–*7*).

Second, incorporating laboratories, hospital infection prevention and control staff, and infectious disease physicians into the EIP sites by actively seeking their support and developing ways to share the information gathered from active surveillance has resulted in truly collaborative networks in each EIP site. Interest is such that in many sites EIP updates are a routine feature of grand rounds in some hospitals. Such interactions have resulted in more efficient and effective networks for communication and data dissemination, more efficient surveillance, and a sense of partnership among many of those involved (e.g., public health professionals, infectious disease clinicians, infection control practitioners, laboratorians) in contributing toward emerging infections work. These networks were used effectively during 2001–2003, before bioterrorism-related preparedness funding became available to support extensive communication systems in all states.

Third, in 2010, the EIPs began to conduct surveillance for health care–associated infections. Addition of capacity in this area has enabled EIP sites to move beyond encouraging hospitals to enroll in the National Healthcare Safety Network and produce annual reports of infection rates by hospital. EIP sites have established systems for ascertaining the number of central line–associated bloodstream infections within their entire catchment populations. Associated validation studies have identified limitations of definitions and enabled more complete case ascertainment. Methods have been established to enable estimation of the total number of nosocomial infections among hospitalized patients, setting the stage for repeated estimation to monitor trends over time (*8*). Interventions have been developed and studied for their effectiveness in some sites through communitywide collaboration.

## Added Value of Academic Center Collaboration

Collaborations with academic health centers have enabled much greater flexibility in the types of surveillance and special studies that the EIPs and, correspondingly, the respective state health departments can undertake. These collaborations not only provide ready access to students looking to participate in research and public health practice projects but also provide easier access for hiring staff for specific short-term projects, making special risk factor studies easier to conduct. In addition, academic center–based staff can conduct intensive surveillance in smaller catchment areas, and interested faculty can collaborate in and enhance population-based surveillance research projects, including tying them into their clinical networks and efforts to seek funding. In Connecticut, for example, faculty from the Yale School of Medicine have taken advantage of, become involved in, and enhanced EIP surveillance for ehrlichiosis, neonatal sepsis, group A GAS disease, chronic liver disease, and precancerous cervical lesions caused by human papillomavirus (HPV) infection; they also have contributed to design and analysis of studies of the effectiveness of pneumococcal, rotavirus, and HPV vaccines. Infectious disease faculty and fellows at the Oregon Health and Science University have contributed to Oregon’s studies of *Clostridium difficile* diarrhea, emerging *Cryptococcus gattii* infections, nontuberculous mycobacterial infections, and surveillance and control of carbapenem-resistant *Enterobacteriaceae*. In Minnesota, collaborations with investigators at the University of Minnesota have enabled studies such as the assessment of variant influenza, matching of antimicrobial-resistant bacteria causing infections in animals with those causing infections in humans, and MRSA infections. In Tennessee, fellows and faculty from the Vanderbilt University School of Medicine have used local EIP data in studies of racial, geographic, and socioeconomic differences in the distribution of pneumococcal serotypes causing invasive disease, group A GAS intracranial infections, invasive pneumococcal infections in patients with sickle cell disease, neonatal early-onset group B *Streptococcus* (GBS) disease, and hospitalizations for influenza. The training relationship established between the Tennessee Department of Health and Vanderbilt University led directly to the prompt recognition and investigation of a recent large, multistate outbreak of fungal meningitis caused by a contaminated injectable steroid product. In Maryland, faculty from Johns Hopkins University designed and led a multisite study using EIP data on risk factors for invasive meningococcal disease among high school students.

## Local Use of Data

Being an EIP site has meant conducting surveillance and obtaining local data for diseases for which the site was not previously conducting surveillance, implementing and evaluating prevention activities that could be or were being used without evaluation by other states, and using the data to reinforce existing or establishing new local disease control guidance. Diseases with new surveillance data for local use have included neonatal GBS and MRSA infections, invasive GAS disease and pneumococcal disease, non-O157 Shiga toxin–producing *Escherichia coli* (STEC), hospitalizations for influenza, *C. difficile* diarrhea (community- and health care–associated), and precancerous lesions caused by HPV infection. Diseases with data that have enabled local reinforcement and enhancement of prevention efforts include neonatal GBS, meningococcal disease, pneumococcal disease, influenza, salmonellosis, and HPV infection. As a result of having and using these data at a local level, EIP sites have become a resource to other states about how such data can be used.

## Site Contributions to the National EIP—Innovation and Synergy

The state-based EIP sites have contributed to the larger EIP in more ways than conducting the agreed-upon surveillance projects and special studies that have provided national-level data leading to new understanding and prevention initiatives on many fronts (*3*). In particular, these sites have been a source of ideas to be considered for new priority EIP projects, multiple and often independent “laboratories” for working out surveillance methods to meet changing needs, an attraction for local academic center staff to become involved and generate spin-off studies, and sources for training of future public health practitioners.

### Innovation

The EIP has a Steering Committee comprising representation from CDC, participating state health departments, and their academic partners from all sites that meets at least annually to discuss administrative matters, progress, and future scientific direction. Although CDC staff usually lead the discussion, goals and priorities are determined collaboratively. Projects originally proposed by EIP sites that have shaped EIP priorities include surveillance for community-associated MRSA (1996 Steering Committee meeting), surveillance for community-associated *C. difficile* infections (2006 FoodNet Steering Committee meeting), and routine analysis of data using area-based socioeconomic measures (2012 Steering Committee meeting). These ideas cut across internal CDC boundaries at the time they were proposed. MRSA and *C. difficile* infections had been largely considered nosocomial problems, housed in CDC’s Division of Healthcare Quality Promotion. Initially, finding the right group at CDC to take an interest in the community perspective proved challenging. Measurement and ongoing monitoring of health conditions and risk factors incorporating measures of socioeconomic status other than race/ethnicity was neither centralized nor a routine concern for most CDC infectious disease programs. As a result, the Steering Committee established a Health Equity Working Group to develop standards and set the agenda for incorporating measures of socioeconomic status into routine EIP surveillance (*9*).

EIP sites also have piloted methods testing the feasibility of conducting population-based surveillance for new conditions and responding to changing laboratory technology. EIP sites piloted various forms of surveillance for community-associated MRSA for several years before settling on a common method (*6*,*7*,*10*,*11*). Collectively, a subset of sites piloted a standardized surveillance method for both community- and hospital-onset *C. difficile* infections, a successful endeavor that resulted in its becoming a core EIP surveillance project (*12*,*13*). Similarly, a subset of EIP sites piloted a standard method for surveillance for precancerous lesions for cervical cancer, demonstrating that the method was feasible. Surveillance for HPV cervical cancer precursors is now a core project for 5 EIP sites (*14*) and is contributing substantially in the assessment of the effectiveness of the vaccine at a population level. When some laboratories stopped performing cultures for *E. coli* O157 and switched to testing for Shiga toxin, the ability to detect outbreaks and monitor trends in *E. coli* O157 was threatened. A pilot project at an EIP site demonstrated the feasibility of turning this crisis into an opportunity to conduct surveillance for both non-O157 and O157 STEC by having the state laboratory culture all Shiga toxin–positive broths into which feces had been inoculated (*15*). Subsequently, surveillance for non-O157 STEC became part of core FoodNet surveillance, and these infections are proving to be even more common than infection by the prototypical *E. coli* O157 strain. Finally, the periodic EIP-sponsored FoodNet Population Surveys have measured frequencies of consumption of a variety of foods, including selected high-risk foods (e.g., alfalfa sprouts, unpasteurized milk). When such data were used in EIP sites as background rates in binomial probability calculations, they enabled rapid identification of food vehicles in outbreaks of salmonellosis, campylobacteriosis, and *E. coli* O157 infection (*16*–*18*). This method, coupled with confirmatory evidence from food tracebacks, case–control studies, or food testing, is now routinely used in many jurisdictions around the country (*19*–*21*).

### Synergy

Collaborations with academic centers also have provided fertile ground for academic researchers to take advantage of the special surveillance projects being conducted in their midst to conduct spin-off projects, sometimes with funding from non-CDC sources. For example, in Connecticut, Yale University researchers have taken advantage of surveillance for ehrlichiosis, GAS, and HPV to conduct special studies beyond those commissioned through the EIP (*22*–*26*). Oregon’s high rates of disease caused by a clone of serogroup B *Neisseria meningitidis* led to a case–control study demonstrating a strong association with exposure to second-hand tobacco smoke (*27*) and to laboratory studies demonstrating the ability of *N. meningitidis* to alter its capsular polysaccharide (*28*). In Minnesota, academic partners have undertaken special studies of *S. aureus* (*29*) and GBS (*30*). In New York (Rochester) and Tennessee, the extent to which EIP surveillance for laboratory-confirmed influenza underestimated influenza-related hospitalizations in children was identified through collaboration and comparison with a research study with a different design than the EIP influenza surveillance (*31*).

## Site-Specific Analyses

EIP sites own their site-specific data and can conduct and publish analyses of these data independently of direct CDC involvement. This ownership has greatly expanded the dissemination of EIP surveillance findings (2 sites alone have published 151 local analyses of data in peer-reviewed publications [Technical Appendix]). In addition, any site wanting to analyze all-site data can make a formal proposal to do so to the Steering Committee which if approved, gives it access to the de-identified all-site dataset (see Technical Appendix for list of multisite publications led by 2 EIP sites). Overall, this flexibility has resulted not only in expanded dissemination of findings but also in expanded analytic creativity and data analysis capacity, and use of data for local and national purposes.

## Training

In another article in this issue, Vugia et al. have summarized the contribution of EIP sites to training of the current and future public health workforce (*32*). Although some training generated by EIP projects has occurred during the course of the CDC-based Epidemic Intelligence Service program and other CDC-based staff have gotten experience with data analysis, most training has occurred at the EIP sites as a result of the partnership in each site with an academic center. In 1 site alone, >190 students received training experiences during 1995–2014 (*32*). Of these, 75 students used their experience to fulfill thesis requirements, and 29 published an article in a peer-reviewed journal.

## Challenges

Although being an EIP site has provided multiple benefits for the state health department and academic center at each site, these benefits have come with some challenges. These challenges include data management; need for frequent human subjects committee reviews of special surveillance and nonsurveillance protocols, often by multiple institutions; and dedicated staff to manage complex budgets and contracts. The funding received by sites does not include the substantial in-kind resources necessary to conduct a large multicomponent program, which also must be integrated with existing public health programs.

EIP sites have found that conducting surveillance and research activities requires attention to the logistics of data acquisition, storage, and distribution. Increasing quantities of data have required development within EIP sites of expanded data storage and handling capacity and increased facility with data systems. Many sites have developed home-grown systems capable of gleaning data electronically, making the data available for epidemiologic analysis, while exporting required fields to CDC for multisite data aggregation. Such systems need built-in flexibility—for example, ready ability to add new conditions or variables of relevance to public health stemming from the sorts of emerging disease problems on which EIPs are called to address. Informatics expertise has proved essential.

In many sites, the EIP is the major source of protocols submitted to institutional review boards (IRBs). Whether a given EIP endeavor constitutes “research” meeting the federal definition (i.e., “designed to develop or contribute to generalizable knowledge” [*33*]) is not always clear because analysis of routinely collected surveillance data may provide knowledge that is, at least in some sense, generalizable. CDC routinely analyzes data generated by state public health agencies in the course of ascertaining and controlling reportable diseases to identify new risk factors and trends that may well be generalizable; not surprisingly, CDC and state health departments often have arrived at different determinations as to whether a given EIP activity constituted research. Moreover, some university collaborators consider any study in which its students are engaged to be research, requiring the protocol’s review by its IRB. The requirement that all IRBs approve the final protocol, and the multiplicity of IRBs (including those of individual hospitals, reviewing and imposing their own requirements on each protocol) for a 10-site EIP study involving university collaboration, can consume considerable time and effort.

With time, activities and expectations for EIPs have expanded, a fact welcomed by most sites. However, funding for the administrative work required by such expansions, including budget, contracts, and IRB tracking, and for hiring experienced epidemiologists to lead new projects has not always kept pace. EIPs note that funding increasingly must be directed to specified projects, leaving them with little flexibility and reduced ability to move beyond collecting data to writing articles for publication or crafting new protocols. As a consequence, such activities are increasingly left to CDC, jeopardizing some of the synergy of the collaborative partnership.

Given the challenges we describe and the frequent necessary coordination of surveillance and epidemiologic activities between local hospitals, laboratories, health departments, and state and federal partners, the structural setup that most EIP sites worked out is one in which the program is located within the lead state health department with or without a co-location within the lead partner school of public health or medicine.

## Summary

The collaborative nature of the EIP has resulted in enhanced surveillance and laboratory capacity and communication networks in the 10 state public health departments. In addition, it has enriched research and public health training at the partner academic centers and produced synergy with the involved CDC programs, broadening the creativity and data analytic and dissemination capacity of all involved entities.

Technical Appendix EIP site-specific peer-reviewed publications, Connecticut and Minnesota, 1996–2014, and published multisite analyses led by staff in EIP sites, Connecticut and Minnesota, 1995-2015.
