# Supplementary material for: Emerging Infections Program—State Health Department Perspective
Source: Emerg Infect Dis. 2015 Sep;21(9):1510–5. doi: 10.3201/eid2109.150428 (PMC4550133; doi:10.3201/eid2109.150428)
Supplement: Technical Appendix — EIP site-specific peer-reviewed publications, Connecticut and Minnesota, 1996–2014, and published multisite analyses led by staff in EIP sites, Connecticut and Minnesota, 1995-2015. [file 15-0428-Techapp-s1.pdf]

Article DOI: http://dx.doi.org/10.3201/eid2109.150428

Emerging Infections Program—State Health Department Perspective

Technical Appendix

EIP Site-Specific Peer-Reviewed Publications, Connecticut and Minnesota, 1996–2014

Connecticut

1. Roberts CL, Morin CA, Addiss DG, Wahlquist SP, Mshar PA, Hadler JL. Factors influencing *Cryptosporidium* testing in Connecticut. J Clin Microbiol. 1996;34:2292–3. PubMed

2. Fiorentino TR, Beall B, Mshar P, Bessen DE. A genetic-based evaluation of the principal tissue reservoir for group A streptococci isolated from normally sterile sites. J Infect Dis. 1997;176:177–82. PubMed http://dx.doi.org/10.1086/514020

3. Morin CA, Roberts CL, Mshar PA, Addiss DG, Hadler JL. What do physicians know about cryptosporidiosis? A survey of Connecticut physicians. Arch Intern Med. 1997;157:1017–22. PubMed http://dx.doi.org/10.1001/archinte.1997.00440300137012

4. Centers for Disease Control and Prevention. Statewide surveillance for ehrlichiosis—Connecticut and New York, 1994–1997. MMWR Morb Mortal Wkly Rep. 1998;47:476–80. PubMed

5. Centers for Disease Control and Prevention. Laboratory practices for prenatal group B streptococcal screening and reporting—Connecticut, Georgia, and Minnesota, 1997–1998 [Connecticut section]. MMWR Morb Mortal Wkly Rep. 1999;48:426–8. PubMed

6. Roome AJ, Hadler JL. Early-onset group B streptococcal disease—United States, 1998–1999 [Connecticut section]. MMWR Morb Mortal Wkly Rep. 2000;49:793–6.

7. IJdo JW, Meek JI, Cartter ML, Magnarelli LA, Wu C, Tenuta SW, et al. The emergence of another tickborne infection in the 12-town area around Lyme, Connecticut: human granulocytic ehrlichiosis. J Infect Dis. 2000;181:1388–93. PubMed http://dx.doi.org/10.1086/315389

8. Centers for Disease Control and Prevention. Adoption of perinatal group B streptococcal disease prevention recommendations by prenatal-care providers—Connecticut and Minnesota, 1998 [Connecticut section]. MMWR Morb Mortal Wkly Rep. 2000;49:228–32. PubMed

9. Morin CA, Hadler JL. Population-based incidence and characteristics of community-onset *Staphylococcus aureus* infections with bacteremia in four metropolitan areas in Connecticut, 1998. J Infect Dis. 2001;184:1029–34. PubMed http://dx.doi.org/10.1086/323459

10. Kluger MD, Sofair AN, Heye CJ, Meek JI, Sodhi RK, Hadler JL. Retrospective validation of a surveillance system for unexplained illness and death: New Haven County, Connecticut. Am J Public Health. 2001;91:1214–9. PubMed http://dx.doi.org/10.2105/AJPH.91.8.1214

11. Chaput EK, Meek JI, Heimer R. Spatial analysis of human granulocytic ehrlichiosis near Lyme, Connecticut. Emerg Infect Dis. 2002;8:943–8. PubMed http://dx.doi.org/10.3201/eid0809.020103

12. Rabatsky-Ehr T, Dingman D, Marcus R, Howard R, Kinney A, Mshar P. Deer meat as the source for a sporadic case of *Escherichia coli* O157:H7 infection, Connecticut. Emerg Infect Dis. 2002;8:525–7. PubMed http://dx.doi.org/10.3201/eid0805.010373

13. Navarro VJ, St Louis T, Bell BP, Sofair AN. Chronic liver disease in the primary care practices of Waterbury, Connecticut. Hepatology. 2003;38:1062. PubMed http://dx.doi.org/10.1002/hep.1840380439

14. Navarro VJ, St Louis TE, Bell BP. Identification of patients with hepatitis C virus infection in New Haven County primary care practices. J Clin Gastroenterol. 2003;36:431–5. PubMed http://dx.doi.org/10.1097/00004836-200305000-00015

15. Trooskin SB, Hadler J, St Louis T, Navarro VJ. Geospatial analysis of hepatitis C in Connecticut: a novel application of a public health tool. Public Health. 2005;119:1042–7. PubMed http://dx.doi.org/10.1016/j.puhe.2005.03.016

16. Centers for Disease Control and Prevention. Laboratory-confirmed non-O157 Shiga toxin–producing *E. coli* surveillance—Connecticut, 2000–2005. MMWR Morb Mortal Wkly Rep. 2007;56:29–31. PubMed

17. Palumbo JP, Meek JI, Fazio DM, Turner SB, Hadler JL, Sofair AN. Unexplained deaths in Connecticut during 2002–2003: failure to consider Category A bioterrorism agents in differential diagnoses. Disaster Med Public Health Prep. 2008;2:87–94. PubMed http://dx.doi.org/10.1097/DMP.0b013e318161315b

18. Vázquez M, Muehlenbein C, Cartter M, Hayes EB, Ertel S, Shapiro ED. Effectiveness of personal protective measures to prevent Lyme disease. Emerg Infect Dis. 2008;14:210–6. PubMed http://dx.doi.org/10.3201/eid1402.070725

19. Marcus R. New information about pediatric foodborne infections: the view from FoodNet. Curr Opin Pediatr. 2008;20:79–84. PubMed http://dx.doi.org/10.1097/MOP.0b013e3282f43067

20. Durante AJ, Meek JI, St Louis T, Navarro VJ, Sofair AN. Quantifying the burden of chronic viral hepatitis–related cirrhosis hospitalizations in New Haven County, Connecticut. Conn Med. 2008;72:393–7. PubMed

21. Durante AJ, St Louis T, Meek JI, Navarro VJ, Sofair AN. The mortality burden of chronic liver disease may be substantially underestimated in the United States. Conn Med. 2008;72:389–92. PubMed

22. Henrich TJ, Lauder N, Desai MM, Sofair AN. Association of alcohol abuse and injection drug use with immunologic and virologic responses to HAART in HIV-positive patients from urban community health clinics. J Community Health. 2008;33:69–77. PubMed http://dx.doi.org/10.1007/s10900-007-9069-1

23. Centers for Disease Control and Prevention. Surveillance for community-associated *C. difficile*—Connecticut, 2006. MMWR Morb Mortal Wkly Rep. 2008;57:340–3. PubMed

24. Wolfson JJ, Jandhyala DM, Gorczyca LA, Qadeer A, Thorpe CM, Manning SD, et al. Prevalence of the operon encoding subtilase cytotoxin in non-O157 STEC isolated from humans in the United States. J Clin Microbiol. 2009;47:3058–9. PubMed http://dx.doi.org/10.1128/JCM.00706-09

25. Sofair AN, Huie-White S, Stabach N, Fine N, Wright R. Use of fax-back surveillance to determine epidemiologic and clinical characteristics of patients diagnosed with hepatitis C in Waterbury, Connecticut. Conn Med. 2009;73:593–5. PubMed

26. Marcus R, Hurd S, Mank L, Mshar P, Phan Q, Jackson K, et al. Chicken salad as the source of a case of *Listeria monocytogenes* infection in Connecticut. J Food Prot. 2009;72:2602–6. PubMed

27. Connally NP, Durante AJ, Yousey-Hindes KM, Meek JI, Nelson RS, Heimer R. Peridomestic Lyme disease prevention: results of a population-based case–control study. Am J Prev Med. 2009;37:201–6. PubMed http://dx.doi.org/10.1016/j.amepre.2009.04.026

28. Backman LA, Melchreit R, Rodriguez R. Validation of the surveillance and reporting of central line–associated bloodstream infection data to a state health department. Am J Infect Control. 2010;38:832–8. PubMed http://dx.doi.org/10.1016/j.ajic.2010.05.016

29. Desai SN, Esposito DB, Shapiro ED, Dennehy PH, Vazquez M. Effectiveness of rotavirus vaccine in preventing hospitalization due to rotavirus gastroenteritis in young children in Connecticut, USA. Vaccine. 2010;28:7501–6. PubMed http://dx.doi.org/10.1016/j.vaccine.2010.09.013

30. Niccolai LM, Mehta NL, Hadler JL. Racial/ethnic and poverty disparities in human papillomavirus vaccination completion. Am J Prev Med. 2011;41:428–33. PubMed http://dx.doi.org/10.1016/j.amepre.2011.06.032

31. Hadler JL, Clogher P, Phan Q, Hurd S, Mandour M, Bemis K, et al. Ten year trends and risk factors for non-O157 Shiga-toxin producing *E. coli* found through Shiga-toxin testing, Connecticut, 2000–2009. Clin Infect Dis. 2011;53:269–76. PubMed http://dx.doi.org/10.1093/cid/cir377

32. Soto K, Petit S, Hadler JL. Changing disparities in invasive pneumococcal disease by socioeconomic status and race in Connecticut, 1998–2008. Public Health Rep. 2011;126(Suppl 3):81–8. PubMed

33. Yousey-Hindes KM, Hadler JL. Neighborhood socioeconomic status and influenza hospitalizations in children: New Haven County, Connecticut, 2003–2010. Am J Public Health. 2011;101:1785–9. PubMed http://dx.doi.org/10.2105/AJPH.2011.300224

34. Niccolai LM, Julian PJ, Bilinski A, Mehta NR, Meek JL, Zelterman D, et al. Geographic poverty and racial/ethnic disparities in cervical cancer precursor rates in Connecticut, 2008–2009. Am J Public Health. 2013;103:156–63. PubMed http://dx.doi.org/10.2105/AJPH.2011.300447

35. Hadler JL, Petit S, Mandour M, Cartter ML. Trends in invasive infection with methicillin-resistant *Staphylococcus aureus*, Connecticut, USA, 2001–2010. Emerg Infect Dis. 2012;18:917–24. PubMed http://dx.doi.org/10.3201/eid1806.120182

36. Mehta NR, Julian PJ, Meek JI, Sosa LE, Bilinski A, Hariri S, et al. Human papillomavirus vaccination history among women with precancerous cervical lesions: disparities and barriers. Obstet Gynecol. 2012;119:575–81. PubMed http://dx.doi.org/10.1097/AOG.0b013e3182460d9f

37. DeCew AE, Hadler JL, Moriarty-Daly A, Niccolai L. The prevalence of HPV associated disease in women under age 21: who will be missed under the new cervical cancer screening guidelines? J Pediatr Adolesc Gynecol. 2013;26:346–9. PubMed http://dx.doi.org/10.1016/j.jpag.2013.06.013

38. Niccolai LM, Julian PJ, Meek JI, McBride V, Hadler JL, Sosa LE. Declining rates of high-grade cervical lesions in young women in Connecticut, USA, 2008–2011. Cancer Epidemiol Biomarkers Prev. 2013;22:1446–50. PubMed

39. Connally NP, Yousey-Hindes K, Meek J. Selection of neighborhood controls for a population-based Lyme disease case–control study by using a commercial marketing database. Am J Epidemiol. 2013;178:276–9. PubMed http://dx.doi.org/10.1093/aje/kws464

40. Niccolai LM, Russ C, Julian PJ, Hariri S, Sinard J, Meek JI, et al. Individual and geographic disparities in human papillomavirus types 16/18 in high-grade cervical lesions: associations with race, ethnicity and poverty. Cancer. 2013;119:3052–8. PubMed http://dx.doi.org/10.1002/cncr.28038

41. Bemis K, Marcus R, Hadler JL. Socioeconomic status and campylobacteriosis, Connecticut, USA, 1999–2009. Emerg Infect Dis. 2014;20:1240–2. PubMed

42. Hadler JL, Yousey-Hindes K, Kudish K, Sacco V, Cartter ML. Evaluation of the initial impact of a requirement for influenza vaccination of children attending licensed childcare or preschool programs—Connecticut 2013. MMWR Morb Mortal Wkly Rep. 2014;63:181–5. PubMed

43. Tam K, Yousey-Hindes K, Hadler JL. Influenza-related hospitalizations of adults associated with low census tract–level socioeconomic status and female sex in New Haven County, CT 2005–2011. Influenza Other Respir Viruses. 2014;8:274–81. http://dx.doi.org/10.1111/irv.12231

44. Niccolai LM, McBride V, Julian PR. Sources of information for assessing human papillomavirus vaccination history among young women. Vaccine. 2014;32:2945–7. PubMed http://dx.doi.org/10.1016/j.vaccine.2014.03.059

45. Waggaman C, Julian P, Niccolai LM. Interactive effects of individual and neighborhood race and ethnicity on rates of high-grade cervical lesions. Cancer Epidemiol. 2014;38:248–52. PubMed http://dx.doi.org/10.1016/j.canep.2014.03.004

Minnesota

1. Bender JB, Hedberg CW, Besser JM, Boxrud DJ, MacDonald KL, Osterholm MT. Surveillance for *Escherichia coli* O157:H7 infections in Minnesota by molecular subtyping. N Engl J Med. 1997;337:388–94. PubMed http://dx.doi.org/10.1056/NEJM199708073370604

2. Smith KE, Besser JM, Hedberg CW, Leano FT, Bender JB, Wicklund JH, et al. Quinolone-resistant *Campylobacter jejuni* infections in Minnesota, 1992–1998. N Engl J Med. 1999;340:1525–32. PubMed http://dx.doi.org/10.1056/NEJM199905203402001

3. Hedberg CW, Angulo FJ, White KE, Langkop CW, Schell WL, Stobierski MG, et al. Outbreaks of salmonellosis associated with eating uncooked tomatoes: implications for public health. Epidemiol Infect. 1999;122:385–93. PubMed http://dx.doi.org/10.1017/S0950268899002393

4. Bender JB, Smith KE, Hedberg C, Osterholm MT. Food-borne disease in the 21st century. What challenges await us? Postgrad Med. 1999;106:109–12, 15–6, 19.

5. Danila RN, Lexau C, Lynfield R, Moore KA, Osterholm MT. Addressing emerging infections. The partnership between public health and primary care physicians. Postgrad Med. 1999;106:90–2, 7–8, 103–5.

6. Centers for Disease Control and Prevention. Intussusception among recipients of rotavirus vaccine—United States, 1998–1999 [Minnesota section]. MMWR Morb Mortal Wkly Rep. 1999;48:577–81. PubMed

7. Centers for Disease Control and Prevention. Four pediatric deaths from community-acquired methicillin-resistant *Staphylococcus aureus*, Minnesota and North Dakota, 1997–1998 [Minnesota section]. MMWR Morb Mortal Wkly Rep. 1999;48:707–10. PubMed

8. Rainbow J, Lynfield R, Johnson JR, Danila RN. Minnesota surveillance for unexplained deaths and critical illnesses of possible infectious cause. Minn Med. 2000;83:51–3. PubMed

9. Deneen VC, Hunt JM, Paule CR, James RI, Johnson RG, Raymond MJ, et al. The impact of foodborne calicivirus disease: the Minnesota experience. J Infect Dis. 2000;181(Suppl 2):S281–3. PubMed http://dx.doi.org/10.1086/315583

10. Centers for Disease Control and Prevention. Adoption of perinatal group B streptococcal disease prevention recommendations by prenatal-care providers—Connecticut and Minnesota, 1998 [Minnesota section]. MMWR Morb Mortal Wkly Rep. 2000;49:228–32. PubMed

11. Bender JB, Hedberg CW, Boxrud DJ, Besser JM, Wicklund JH, Smith KE, et al. Use of molecular subtyping in surveillance for *Salmonella enterica* serotype Typhimurium. N Engl J Med. 2001;344:189–95. PubMed http://dx.doi.org/10.1056/NEJM200101183440305

12. Ehresmann KR, Ramesh A, Como-Sabetti K, Peterson DC, Whitney CG, Moore KA. Factors associated with self-reported pneumococcal immunization among adults 65 years of age or older in the Minneapolis–St. Paul metropolitan area. Prev Med. 2001;32:409–15. PubMed http://dx.doi.org/10.1006/pmed.2001.0839

13. Naimi T, Ringwald P, Besser R, Thompson S. Antimicrobial resistance. Emerg Infect Dis. 2001;7(Suppl):548. PubMed http://dx.doi.org/10.3201/eid0707.017727

14. Hedberg CW, Smith KE, Besser JM, Boxrud DJ, Hennessy TW, Bender JB, et al. Limitations of pulsed-field gel electrophoresis for the routine surveillance of *Campylobacter* infections. J Infect Dis. 2001;184:242–4. PubMed http://dx.doi.org/10.1086/322005

15. Centers for Disease Control and Prevention. Update: unexplained deaths following knee surgery—Minnesota, November 2001. MMWR Morb Mortal Wkly Rep. 2001;50:1035–6. PubMed

16. Centers for Disease Control and Prevention. Unexplained deaths following knee surgery—Minnesota, 2001. MMWR Morb Mortal Wkly Rep. 2001;50:1080. PubMed

17. Groom AV, Wolsey DH, Naimi TS, Smith K, Johnson S, Boxrud D, et al. Community-acquired methicillin-resistant *Staphylococcus aureus* in a rural American Indian community. JAMA. 2001;286:1201–5. PubMed http://dx.doi.org/10.1001/jama.286.10.1201

18. Naimi TS, LeDell KH, Boxrud DJ, Groom AV, Steward CD, Johnson SK, et al. Epidemiology and clonality of community-acquired methicillin-resistant *Staphylococcus aureus* in Minnesota, 1996–1998. Clin Infect Dis. 2001;33:990–6. PubMed http://dx.doi.org/10.1086/322693

19. Naimi TS, Anderson D, O'Boyle C, Boxrud DJ, Johnson SK, Tenover FC, et al. Vancomycin-intermediate *Staphylococcus aureus* with phenotypic susceptibility to methicillin in a patient with recurrent bacteremia. Clin Infect Dis. 2003;36:1609–12. PubMed http://dx.doi.org/10.1086/375228

20. Johnson JR, Clabots C, Azar M, Boxrud DJ, Besser JM, Thurn JR. Molecular analysis of a hospital cafeteria-associated salmonellosis outbreak using modified repetitive element PCR fingerprinting. J Clin Microbiol. 2001;39:3452–60. PubMed http://dx.doi.org/10.1128/JCM.39.10.3452-3460.2001

21. Bartkus JM, Juni BA, Ehresmann K, Miller CA, Sanden GN, Cassiday PK, et al. Identification of a mutation associated with erythromycin resistance in *Bordetella pertussis*: implications for surveillance of antimicrobial resistance. J Clin Microbiol. 2003;41:1167–72. PubMed http://dx.doi.org/10.1128/JCM.41.3.1167-1172.2003

22. Naimi TS, Wicklund JH, Olsen SJ, Krause G, Wells JG, Bartkus JM, et al. Concurrent outbreaks of *Shigella sonnei* and enterotoxigenic *Escherichia coli* infections associated with parsley: implications for surveillance and control of foodborne illness. J Food Prot. 2003;66:535–41. PubMed

23. Naimi TS, Anderson D, O'Boyle C, Boxrud DJ, Johnson SK, Tenover FC, et al. Vancomycin-intermediate *Staphylococcus aureus* with phenotypic susceptibility to methicillin in a patient with recurrent bacteremia. Clin Infect Dis. 2003;36:1609–12. PubMed http://dx.doi.org/10.1086/375228

24. Ogunmodede F, Virnig BA, Danila R, Lynfield R. Prevention of perinatal group B streptococcal disease in Minnesota: results from a retrospective cohort study and new prevention guidelines. Minn Med. 2003;86:40–5. PubMed

25. Johnson JR, Murray AC, Gajewski A, Sullivan M, Snippes P, Kuskowski MA, et al. Isolation and molecular characterization of nalidixic acid–resistant extraintestinal pathogenic *Escherichia coli* from retail chicken products. Antimicrob Agents Chemother. 2003;47:2161–8. PubMed http://dx.doi.org/10.1128/AAC.47.7.2161-2168.2003

26. Naimi TS, LeDell KH, Como-Sabetti K, Borchardt SM, Boxrud DJ, Etienne J, et al. Comparison of community- and health care–associated methicillin-resistant *Staphylococcus aureus* infection. JAMA. 2003;290:2976–84. PubMed http://dx.doi.org/10.1001/jama.290.22.2976

27. Bender JB, Shulman SA. Reports of zoonotic disease outbreaks associated with animal exhibits and availability of recommendations for preventing zoonotic disease transmission from animals to people in such settings. J Am Vet Med Assoc. 2004;224:1105–9. PubMed http://dx.doi.org/10.2460/javma.2004.224.1105

28. Smith KE, Stenzel SA, Bender JB, Wagstrom E, Soderlund D, Leano FT, et al. Outbreaks of enteric infections caused by multiple pathogens associated with calves at a farm day camp. Pediatr Infect Dis J. 2004;23:1098–104. PubMed

29. Ogunmodede F, Jones JL, Scheftel J, Kirkland E, Schulkin J. Listeriosis prevention knowledge among pregnant women in the USA. Infect Dis Obstet Gynecol. 2005;13:11–5. PubMed http://dx.doi.org/10.1155/2005/734814

30. Johnson JR, Delavari P, O’Bryan TT, Smith KE, Tatini S. Contamination of retail foods, particularly turkey, from community markets (Minnesota, 1999–2000) with antimicrobial-resistant and extraintestinal pathogenic *Escherichia coli*. Foodborne Pathog Dis. 2005;2:38–49. PubMed http://dx.doi.org/10.1089/fpd.2005.2.38

31. Johnson JR, Kuskowski MA, Smith K, O’Bryan TT, Tatini S. Antimicrobial-resistant and extraintestinal pathogenic *Escherichia coli* in retail foods. J Infect Dis. 2005;191:1040–9. PubMed http://dx.doi.org/10.1086/428451

32. Kiang KM, Kieke BA, Como-Sabetti K, Lynfield R, Besser RE, Belongia E. Clinician knowledge and beliefs after statewide program to promote appropriate antimicrobial drug use. Emerg Infect Dis. 2005;11:904–11. PubMed http://dx.doi.org/10.3201/eid1106.050144

33. Laine ES, Scheftel JM, Boxrud DJ, Vought KJ, Danila RN, Elfering KM, et al. Outbreak of *Escherichia coli* O157:H7 infections associated with nonintact blade-tenderized frozen steaks sold by door-to-door vendors. J Food Prot. 2005;68:1198–202. PubMed

34. Rainbow J, Cebelinski E, Bartkus J, Glennen A, Boxrud D, Lynfield R. Rifampin-resistant meningococcal disease. Emerg Infect Dis. 2005;11:977–9. PubMed http://dx.doi.org/10.3201/eid1106.050143

35. Smith KE, Anderson F, Medus C, Leano F, Adams J. Outbreaks of salmonellosis at elementary schools associated with dissection of owl pellets. Vector Borne Zoonotic Dis. 2005;5:133–6. PubMed http://dx.doi.org/10.1089/vbz.2005.5.133

36. Morin CA, White K, Schuchat A, Danila R, Lynfield R. Perinatal group B streptococcal disease prevention, Minnesota. Emerg Infect Dis. 2005;11:1467–9. PubMed http://dx.doi.org/10.3201/eid1109.041109

37. Buck JM, Como-Sabetti K, Harriman KH, Danila RN, Boxrud DJ, Glennen A, et al. Community-associated methicillin-resistant *Staphylococcus aureus* infections: Minnesota, 2000–2003. Emerg Infect Dis. 2005;11:1532–8. PubMed http://dx.doi.org/10.3201/eid1110.050141

38. Wedel SD, Bender JB, Leano FT, Boxrud D, Hedberg C, Smith KE. Antimicrobial susceptibility and pulsed-field gel electrophoresis profiles of clinical *Salmonella* Typhimurium isolates of human and animal origin in Minnesota, 1997–2003. Emerg Infect Dis. 2005;11:1899–906. PubMed http://dx.doi.org/10.3201/eid1112.050158

39. Kiang KM, Scheftel JM, Leano FT, Taylor CM, Belle-Isle PA, Cebelinski EA, et al. Recurrent outbreaks of cryptosporidiosis associated with calves among students at an educational farm programme, Minnesota, 2003. Epidemiol Infect. 2006;134:878–86. PubMed http://dx.doi.org/10.1017/S0950268805005649

40. Liesener AL, Smith KE, Davis RD, Bender JB, Danila RN, Neitzel DF, et al. Circumstances of bat encounters and knowledge of rabies among Minnesota residents submitting bats for rabies testing. Vector Borne Zoonotic Dis. 2006;6:208–15. PubMed http://dx.doi.org/10.1089/vbz.2006.6.208

41. Medus C, Smith KE, Bender JB, Besser JM, Hedberg CW. *Salmonella* outbreaks in restaurants in Minnesota, 1995 through 2003: evaluation of the role of infected foodworkers. J Food Prot. 2006;69:1870–8. PubMed

42. Buck JM, Lexau C, Shapiro M, Glennen A, Boxrud D, Koziol B, et al. Community outbreak of conjunctivitis caused by nontypeable *Streptococcus pneumoniae* in Minnesota. Pediatr Infect Dis J. 2006;25:906–11. PubMed http://dx.doi.org/10.1097/01.inf.0000238143.96607.ec

43. Swanson SJ, Neitzel D, Reed KD, Belongia EA. Coinfections acquired from *Ixodes* ticks. Clin Microbiol Rev. 2006;19:708–27. PubMed http://dx.doi.org/10.1128/CMR.00011-06

44. Como-Sabetti K, Harriman K, Juni B, Westbrook A, Cebelinski E, Boxrud D, et al. Methicillin resistant *Staphylococcus aureus* at canoe camp. Emerg Infect Dis. 2006;12:1759–61. PubMed http://dx.doi.org/10.3201/eid1211.060363

45. Swanson SJ, Snider C, Braden CR, Boxrud D, Wunschmann A, Rudroff JA, et al. Multidrug-resistant *Salmonella enterica* serotype Typhimurium associated with pet rodents. N Engl J Med. 2007;356:21–8. PubMed http://dx.doi.org/10.1056/NEJMoa060465

46. Boxrud D, Pederson-Gulrud K, Wotton J, Medus C, Lyszkowicz E, Besser J, et al. Comparison of multiple-locus variable-number tandem repeat analysis, pulsed-field gel electrophoresis, and phage typing for subtype analysis of *Salmonella enterica* serotype Enteritidis. J Clin Microbiol. 2007;45:536–43. PubMed http://dx.doi.org/10.1128/JCM.01595-06

47. Gahr P, Harper J, Kieke B Jr, Como-Sabetti K, Craig Christianson R, Williams D, et al. Healthcare professional surveys: judicious antibiotic use in Minnesota long-term care facilities. J Am Geriatr Soc. 2007;55:473–4. PubMed http://dx.doi.org/10.1111/j.1532-5415.2007.01080.x

48. Jungk J, Como-Sabetti K, Stinchfield P, Ackerman P, Harriman K. Epidemiology of methicillin-resistant *Staphylococcus aureus* at a pediatric healthcare system, 1991–2003. Pediatr Infect Dis J. 2007;26:339–44. PubMed http://dx.doi.org/10.1097/01.inf.0000257452.58182.e1

49. Johnson JR, Sannes MR, Croy C, Johnson B, Clabots C, Kuskowski MA, et al. Antimicrobial drug-resistant *Escherichia coli* from humans and poultry products, Minnesota and Wisconsin, 2002–2004. Emerg Infect Dis. 2007;13:838–46. PubMed http://dx.doi.org/10.3201/eid1306.061576

50. O'Fallon E, Harper J, Shaw S, Lynfield R. Antibiotic and infection tracking in Minnesota long-term care facilities. J Am Geriatr Soc. 2007;55:1243–7. PubMed http://dx.doi.org/10.1111/j.1532-5415.2007.01247.x

51. Triden L, Glennen A, Juni B, Lynfield R. Invasive *Haemophilus influenzae* disease and antibiotic susceptibility of invasive isolates in Minnesota, 2002–2005. Infect Dis Clin Pract. 2007;15:373–6. http://dx.doi.org/10.1097/IPC.0b013e318157d260

52. Sannes MR, Belongia EA, Kieke B, Smith K, Kieke A, Vandermause M, et al. Predictors of antimicrobial-resistant *Escherichia coli* in the feces of vegetarians and newly hospitalized adults in Minnesota and Wisconsin. J Infect Dis. 2008;197:430–4. PubMed http://dx.doi.org/10.1086/525530

53. Centers for Disease Control and Prevention. Emergence of fluoroquinolone-resistant *Neisseria meningitidis*—Minnesota and North Dakota, 2007–2008 [Minnesota section]. MMWR Morb Mortal Wkly Rep. 2008;57:173–5. PubMed

54. Lexau CA. Changing epidemiology of pneumococcal pulmonary disease in the era of the heptavalent vaccine. Curr Infect Dis Rep. 2008;10:229–35. PubMed http://dx.doi.org/10.1007/s11908-008-0038-3

55. Buck JM, Harriman KH, Juni BA, Gall K, Boxrud DJ, Glennen A, et al. No change in methicillin-resistant *Staphylococcus aureus* nasal colonization rates among Minnesota school childrens during 2 study periods. Infect Dis Clin Pract. 2008;16:163–5. http://dx.doi.org/10.1097/IPC.0b013e318168ff48

56. Rainbow J, Jewell B, Danila RN, Boxrud D, Beall B, Van Beneden C, et al. Invasive group A streptococcal disease in nursing homes, Minnesota, 1995–2006. Emerg Infect Dis. 2008;14:772–7. PubMed

57. Fuller CC, Jawahir SL, Leano FT, Bidol SA, Signs K, Davis C, et al. Multi-state *Salmonella* Typhimurium outbreak associated with frozen vacuum-packed rodents used to feed snakes. Zoonoses Public Health. 2008;55:481–7. PubMed

58. Smith KE, Medus C, Meyer SD, Boxrud DJ, Leano F, Hedberg CW, et al. Outbreaks of salmonellosis in Minnesota (1998 to 2006) associated with frozen, microwaveable, breaded, stuffed chicken products. J Food Prot. 2008;71:2153–60. PubMed

59. Como-Sabetti K, Harriman KH, Buck JM, Glennen A, Boxrud DJ, Lynfield R. Community-associated methicillin-resistant *Staphylococcus aureus*: trends in case and isolate characteristics from six years of prospective surveillance. Public Health Rep. 2009;124:427–35. PubMed

60. Lesher L, DeVries A, Danila R, Lynfield R. Evaluation of surveillance methods for staphylococcal toxic shock syndrome. Emerg Infect Dis. 2009;15:770–3. PubMed http://dx.doi.org/10.3201/eid1505.080826

61. Hedican EB, Medus C, Besser JM, Juni BA, Koziol B, Taylor C, et al. Characteristics of O157 versus non-O157 Shiga toxin–producing *Escherichia coli* infections in Minnesota, 2000–2006. Clin Infect Dis. 2009;49:358–64. PubMed http://dx.doi.org/10.1086/600302

62. Kothari NJ, Morin CA, Glennen A, Jackson D, Harper J, Schrag SJ, et al. Invasive group B streptococcal disease in the elderly in Minnesota, USA, 2003–2007. Emerg Infect Dis. 2009;15:1279–81. PubMed http://dx.doi.org/10.3201/eid1508.081381

63. Hedican E, Hooker C, Jenkins T, Medus C, Jawahir S, Leano F, et al. Restaurant *Salmonella* Enteritidis outbreak associated with an asymptomatic infected food worker. J Food Prot. 2009;72:2332–6. PubMed

64. Holzbauer SM, DeVries AS, Sejvar JJ, Lees CH, Adjemian J, McQuiston JH, et al. Epidemiologic investigation of immune-mediated polyradiculoneuropathy among abattoir workers exposed to porcine brain. PLoS ONE. 2010;5:e9782. PubMed http://dx.doi.org/10.1371/journal.pone.0009782

65. Robinson TJ, Cebelinski EA, Taylor C, Smith KE. Evaluation of the positive predictive value of rapid assays used by clinical laboratories in Minnesota for the diagnosis of cryptosporidiosis. Clin Infect Dis. 2010;50:e53–5. PubMed http://dx.doi.org/10.1086/651423

66. Boxrud D, Monson T, Stiles T, Besser J. The role, challenges, and support of Pulsenet laboratories in detecting foodborne disease outbreaks. Public Health Rep. 2010;125(Suppl 2):57–62. PubMed

67. Holzbauer SM, Kemperman MM, Lynfield R. Death due to community-associated *Clostridium difficile* in a woman receiving prolonged antibiotic therapy for suspected Lyme disease. Clin Infect Dis. 2010;51:369–70. PubMed http://dx.doi.org/10.1086/654808

68. Hedican E, Miller B, Ziemer B, Lemaster P, Jawahir S, Leano F, et al. Salmonellosis outbreak due to chicken contact leading to a foodborne outbreak associated with infected delicatessen workers. Foodborne Pathog Dis. 2010;7:995–7. PubMed http://dx.doi.org/10.1089/fpd.2009.0495

69. Rounds JM, Hedberg CW, Meyer S, Boxrud D, Smith KE. *Salmonella nterica* pulsed-field gel electrophoresis clusters, Minnesota, USA, 2001–2007. Emerg Infect Dis. 2010;16:1678–85. PubMed http://dx.doi.org/10.3201/eid1611.100368

70. Medus C, Smith KE, Bender JB, Leano F, Hedberg CW. *Salmonella* infections in food workers identified through public health surveillance in Minnesota: impact on outbreak recognition. J Food Prot. 2010;73:2053–8. PubMed

71. Li J, Smith K, Kaehler D, Everstine K, Rounds J, Hedberg C. Evaluation of a statewide foodborne illness complaint surveillance system in Minnesota. J Food Prot. 2010;73:2059–64. PubMed

72. Scheftel JM, Griffith JM, Leppke BA, Pantlin GC, Snippes PM, Wunschmann A. Tularaemia in Minnesota: case report and brief epidemiology. Zoonoses Public Health. 2010;57:e165–9. PubMed http://dx.doi.org/10.1111/j.1863-2378.2009.01318.x

73. Li J, Maclehose R, Smith K, Kaehler D, Hedberg C. Development of a *Salmonella* screening tool for consumer complaint–based foodborne illness surveillance systems. J Food Prot. 2011;74:106–10. PubMed http://dx.doi.org/10.4315/0362-028X.JFP-10-312

74. Como-Sabetti KJ, Harriman KH, Fridkin SK, Jawahir SL, Lynfield R. Risk factors for community-associated *Staphylococcus aureus* infections: results from parallel studies including methicillin-resistant and methicillin-sensitive *S. aureus* compared to uninfected controls. Epidemiol Infect. 2011;139:419–29. PubMed http://dx.doi.org/10.1017/S0950268810001111

75. Lees CH, Avery C, Asherin R, Rainbow J, Danila R, Smelser C, et al. Pandemic (H1N1) 2009–associated deaths detected by unexplained death and medical examiner surveillance. Emerg Infect Dis. 2011;17:1479–83. PubMed

76. DeVries AS, Lesher L, Schlievert PM, Rogers T, Villaume LG, Danila R, et al. Staphylococcal toxic shock syndrome 2000–2006: epidemiology, clinical features, and molecular characteristics. PLoS ONE. 2011;6:e22997. PubMed http://dx.doi.org/10.1371/journal.pone.0022997

77. Nerby JM, Gorwitz R, Lesher L, Juni B, Jawahir S, Lynfield R, et al. Risk factors for household transmission of community-associated methicillin-resistant *Staphylococcus aureus*. Pediatr Infect Dis J. 2011;30:927–32. PubMed http://dx.doi.org/10.1097/INF.0b013e31822256c3

78. Lowther SA, Medus C, Scheftel J, Leano F, Jawahir S, Smith K. Foodborne outbreak of *Salmonella* subspecies IV infections associated with contamination from bearded dragons. Zoonoses Public Health. 2011;58:560–6. PubMed http://dx.doi.org/10.1111/j.1863-2378.2011.01403.x

79. Smith KE, Wilker PR, Reiter PL, Hedican EB, Bender JB, Hedberg CW. Antibiotic treatment of *Escherichia coli* O157 infection and the risk of hemolytic uremic syndrome, Minnesota. Pediatr Infect Dis J. 2012;31:37–41. PubMed http://dx.doi.org/10.1097/INF.0b013e31823096a8

80. Rounds JM, Rigdon CE, Muhl LJ, Forstner M, Danzeisen GT, Koziol BS, et al. Non-O157 Shiga toxin–producing *Escherichia coli* infections associated with venison. Emerg Infect Dis. 2012;18:279–82. PubMed http://dx.doi.org/10.3201/eid1802.110855

81. Bender JB, Waters KC, Nerby J, Olsen KE, Jawahir S. Methicillin-resistant *Staphylococcus aureus* (MRSA) isolated from pets living in households with MRSA-infected children. Clin Infect Dis. 2012;54:449–50. PubMed http://dx.doi.org/10.1093/cid/cir714

82. Miller BD, Rigdon CE, Ball J, Rounds J, Klos R, Brennan BM, et al. Use of trace-back methods to confirm the source of a multi-state *E. coli* O157:H7 outbreak due to in-shell hazelnuts. J Food Prot. 2012;75:320–7. PubMed http://dx.doi.org/10.4315/0362-028X.JFP-11-309

83. Lowther SA, Shinoda N, Juni BA, Theodore MJ, Wang X, Jawahir SL, et al. *Haemophilus influenzae* type b infection, vaccination, and *H. influenzae* carriage in children in Minnesota, 2008–2009. Epidemiol Infect. 2012;140:566–74. PubMed http://dx.doi.org/10.1017/S0950268811000793

84. Koene R, Boulware DR, Kemperman M, Konety SH, Groth M, Jesserun J, et al. Acute heart failure from Lyme carditis. Circ Heart Fail. 2012;5:e24–6. PubMed http://dx.doi.org/10.1161/CIRCHEARTFAILURE.111.965533

85. Rounds JM, Boxrud DJ, Jawahir SL, Smith KE. Dynamics of *Escherichia coli* O157:H7 outbreak detection and investigation, Minnesota 2000–2008. Epidemiol Infect. 2012;140:1430–8. PubMed http://dx.doi.org/10.1017/S0950268811002330

86. DeVries A, Wotton J, Lees C, Boxrud D, Uyeki T, Lynfield R. Neuraminidase H275Y and hemagglutinin D222G mutations in a fatal case of pandemic influenza A (H1N1) virus infection. Influenza Other Respir Viruses. 2012;6:e85–8. PubMed http://dx.doi.org/10.1111/j.1750-2659.2011.00329.x

87. Spaulding AB, Radi D, Macleod H, Lynfield R, Larson M, Hyduke T, et al. Design and implementation of a statewide influenza nurse triage line in response to pandemic H1N1 influenza. Public Health Rep. 2012;127:532–40. PubMed

88. Spaulding AB, Radi D, Macleod H, Lynfield R, Larson M, Hyduke T, et al. Satisfaction and public health cost of a statewide influenza nurse triage line in response to pandemic H1N1 influenza. PLoS ONE. 2013;8:e50492. PubMed http://dx.doi.org/10.1371/journal.pone.0050492

89. Fowler HN, Brown P, Rovira A, Shade P, Klammer K, Smith K, et al. *Streptococcus suis* meningitis in swine worker, Minnesota, USA. Emerg Infect Dis. 2013;19:330–1. PubMed http://dx.doi.org/10.3201/eid1902.120918

90. Ferrieri P, Lynfield R, Creti R, Flores AE. Serotype IV and invasive group B *Streptococcus* disease in neonates, Minnesota, USA, 2000–2010. Emerg Infect Dis. 2013;19:551–8. PubMed

91. Miller BD, Rigdon CE, Robinson TJ, Hedberg C, Smith KE. Use of global trade item numbers in the investigation of a *Salmonella* Newport outbreak associated with blueberries in Minnesota, 2010. J Food Prot. 2013;76:762–9. PubMed http://dx.doi.org/10.4315/0362-028X.JFP-12-407

92. Saupe AA, Kaehler D, Cebelinski EA, Nefzger B, Hall AJ, Smith KE. Norovirus surveillance among callers to foodborne illness complaint hotline, Minnesota, USA, 2011–2013. Emerg Infect Dis. 2013;19:1293–6. PubMed http://dx.doi.org/10.3201/eid1908.130462

93. Kemble SK, Westbrook A, Lynfield R, Bogard A, Koktavy N, Gall K, et al. Foodborne outbreak of group A *Streptococcus* pharyngitis associated with a high school dance team banquet—Minnesota, 2012. Clin Infect Dis. 2013;57:648–54. PubMed http://dx.doi.org/10.1093/cid/cit359

94. Bogard AK, Fuller CC, Radke V, Selman CA, Smith KE. Ground beef handling and cooking practices in restaurants in eight states. J Food Prot. 2013;76:2132–40. PubMed http://dx.doi.org/10.4315/0362-028X.JFP-13-126

95. Robinson TJ, Scheftel JM, Smith KE. Raw milk consumption among patients with non–outbreak-related enteric infections, Minnesota, USA, 2001–2010. Emerg Infect Dis. 2014;20:38–44. PubMed

96. Lynfield R, Schaffner W. Can we conquer coqueluche? J Infect Dis. 2014;209(Suppl 1):S1–3. PubMed http://dx.doi.org/10.1093/infdis/jit487

97. Kenyon C, Banerjee E, Sweet K, Miller C, Ehresmann K. Assessing the impact of a pertussis active surveillance program on provider testing behavior, Minnesota 2005–2009. Am J Public Health. 2014;104:e34–9. PubMed http://dx.doi.org/10.2105/AJPH.2013.301815

98. Pereira EC, Shaw KM, Vagnone PM, Harper JE, Kallen AJ, Limbago BM, et al. Thirty-day laboratory-based surveillance for carbapenem-resistant *Enterobacteriaceae* in the Minneapolis–St. Paul metropolitan area. Infect Control Hosp Epidemiol. 2014;35:423–5. PubMed http://dx.doi.org/10.1086/675602

99. Shaw KM, Harper JE, Vagnone PM, Lynfield R. Establishing surveillance for carbapenem-resistant *Enterobacteriaceae* in Minnesota, 2012. Infect Control Hosp Epidemiol. 2014;35:451–3. PubMed http://dx.doi.org/10.1086/675615

100. Whitten T, Bender JB, Smith K, Leano F, Scheftel J. Reptile-associated salmonellosis in Minnesota, 1996–2011. Zoonoses Public Health. 2015;62:199–208. PubMed http://dx.doi.org/10.1111/zph.12140

101. Klumb C, Saunders S, Smith K. *E. coli* O157:H7 surveillance in agricultural populations in Minnesota. Journal of Agromedicine. 2014;19:221. http://dx.doi.org/10.1080/1059924X.2014.890555

102. Choi MJ, Morin CA, Scheftel J, Vetter SM, Smith K, Lynfield R, et al. Variant influenza associated with live animal markets, Minnesota. Zoonoses Public Health. 2014. Epub ahead of print. PubMed http://dx.doi.org/10.1111/zph.12139

103. Holzbauer SM, Agger WA, Hall RL, Johnson GM, Schmitt D, Garvey A, et al. Outbreak of *Trichinella spiralis* infections associated with consumption of wild boar hunted at a game farm in Iowa. Clin Infect Dis. 2014;59:1750–6. PubMed http://dx.doi.org/10.1093/cid/ciu713

104. Robinson SJ, Neitzel DF, Moen RA, Craft ME, Hamilton KE, Johnson LB, et al. Disease risk in a dynamic environment: the spread of tick-borne pathogens in Minnesota, USA. EcoHealth. 2015;12:152–63. PubMed http://dx.doi.org/10.1007/s10393-014-0979-y

105. Danila RN, Eikmeier D, Robinson T, La Pointe A, DeVries A. Two concurrent enteric disease outbreaks among men who have sex with men, Minneapolis–St. Paul area. Clin Infect Dis. 2014;59:987–9. PubMed http://dx.doi.org/10.1093/cid/ciu478

106. Fowler H, Scheftel J. Survey of occupational hazards in veterinary medicine, Minnesota 2012. J Am Vet Med Assoc. In press.

Published Multisite Analyses Led by Staff in EIP sites, Connecticut and Minnesota, 1995–2015

Connecticut

1. Baltimore RS, Huie SM, Meek JI, Schuchat A, O'Brien KL. Early-onset neonatal sepsis in the era of group B streptococcal prevention. Pediatrics. 2001;108:1094–8. PubMed http://dx.doi.org/10.1542/peds.108.5.1094

2. Vanden Eng J, Marcus R, Hadler JL, Imhoff B, Vugia DJ, Cieslak P, et al. Consumer attitudes and use of antibiotics. Emerg Infect Dis. 2003;9:1128–35. PubMed http://dx.doi.org/10.3201/eid0909.020591

3. Marcus R, Fiorentino T, Mohle-Boetani J, Farley MM, Medus C, Shiferaw B, et al. Dramatic decrease in S*almonella* serotype Enteritidis (SE) in 5 FoodNet sites. 1996–1999. Clin Infect Dis. 2004;38(Suppl 3):S135–41. PubMed http://dx.doi.org/10.1086/381579

4. Heffernan RT, Barrett NL, Gallagher K, Hadler J, Harrison L, Reingold A, et al. Declining invasive *Streptococcus pneumoniae* infections among people living with AIDS in the era of highly-active antiretroviral therapy (HAART), 1995–2000. J Infect Dis. 2005;191:2038–45. PubMed http://dx.doi.org/10.1086/430356

5. Sofair AN, Lyon GM, Huie-White S, Reiss E, Harrison LH, Sanza LT, et al. Epidemiology of community-onset candidemia in Connecticut and Maryland. Clin Infect Dis. 2006;43:32–9. PubMed http://dx.doi.org/10.1086/504807

6. Marcus R, Varma JK, Medus C, Boothe EJ, Anderson BJ, Crume T, et al. Re-assessment of risk factors for sporadic *Salmonella* serotype Enteritidis infections: a case–control study in five FoodNet sites. 2002–2003. Epidemiol Infect. 2007;135:84–92. PubMed http://dx.doi.org/10.1017/S0950268806006558

7. Ferrucci LM, Bell BP, Dhotre KB, Manos MM, Terrault NA, Zaman A, et al. Complementary and alternative medicine use in chronic liver disease patients. J Clin Gastroenterol. 2010;44:e40–5. PubMed http://dx.doi.org/10.1097/MCG.0b013e3181b766ed

8. Sofair AN, Barry V, Manos MM, Thomas A, Zaman A, Terrault NA, et al. The epidemiology and clinical characteristics of patients with newly diagnosed alcohol-related liver disease: results from population-based surveillance. J Clin Gastroenterol. 2010;44:301–7. PubMed http://dx.doi.org/10.1097/MCG.0b013e3181b3f760

9. Hurd S, Patrick M, Hatch J, Clogher P, Wymore K, Cronquist AB, et al. Clinical laboratory practices for the isolation and identification of *Campylobacter* in Foodborne Diseases Active Surveillance Network (FoodNet) sites: baseline information for understanding changes in surveillance data. Clin Infect Dis. 2012;54(Suppl 5):S440–5. PubMed http://dx.doi.org/10.1093/cid/cis245

10. Clogher P, Hurd S, Hoefer D, Hadler J, Pasutti L, Cosgrove S, et al. Assessment of physician knowledge and practices concerning STEC infection and enteric illness, 2009, FoodNet. Clin Infect Dis. 2012;54(suppl 5):S446–52. PubMed http://dx.doi.org/10.1093/cid/cis246

Minnesota

1. Kassenborg HD, Hedberg CW, Hoekstra M, Evans MC, Chin AE, Marcus R, et al. Farm visits and undercooked hamburgers as major risk factors for sporadic *Escherichia coli* O157:H7 infection: data from a case–control study in 5 FoodNet sites. Clin Infect Dis. 2004;38(Suppl 3):S271–8. PubMed http://dx.doi.org/10.1086/381596

2. Kassenborg HD, Smith KE, Vugia DJ, Rabatsky-Ehr T, Bates MR, Carter MA, et al. Fluoroquinolone-resistant *Campylobacter* infections: eating poultry outside of the home and foreign travel are risk factors. Clin Infect Dis. 2004;38(Suppl 3):S279–84. PubMed http://dx.doi.org/10.1086/381597

3. Bender JB, Smith KE, McNees AA, Rabatsky-Ehr TR, Segler SD, Hawkins MA, et al. Factors affecting surveillance data on *Escherichia coli* O157 infections collected from FoodNet sites, 1996–1999. Clin Infect Dis. 2004;38(Suppl 3):S157–64. PubMed http://dx.doi.org/10.1086/381582

4. Nelson JM, Smith KE, Vugia DJ, Rabatsky-Ehr T, Segler SD, Kassenborg HD, et al. Prolonged diarrhea due to ciprofloxacin-resistant *Campylobacter* infection. J Infect Dis. 2004;190:1150–7. PubMed http://dx.doi.org/10.1086/423282

5. Lexau CA, Lynfield R, Danila R, Pilishvili T, Facklam R, Farley MM, et al. Changing epidemiology of invasive pneumococcal disease among older adults in the era of pediatric pneumococcal conjugate vaccine. JAMA. 2005;294:2043–51. PubMed http://dx.doi.org/10.1001/jama.294.16.2043

6. Castor ML, Whitney CG, Como-Sabetti K, Facklam RR, Ferrieri P, Bartkus JM, et al. Antibiotic resistance patterns in invasive group B streptococcal isolates. Infect Dis Obstet Gynecol. 2008;2008:727505.
